# Orchid B_sister_ gene *PeMADS28* displays conserved function in ovule integument development

**DOI:** 10.1038/s41598-020-79877-9

**Published:** 2021-01-13

**Authors:** Ching-Yu Shen, You-Yi Chen, Ke-Wei Liu, Hsiang-Chia Lu, Song-Bin Chang, Yu-Yun Hsiao, Fengxi Yang, Genfa Zhu, Shuang-quan Zou, Lai-Qiang Huang, Zhong-Jian Liu, Wen-Chieh Tsai

**Affiliations:** 1 Institute of Tropical Plant Sciences and Microbiology, National Cheng Kung University, Tainan, 701 Taiwan; 2 Department of Life Sciences, National Cheng Kung University, Tainan, 701 Taiwan; 3 Center for Biotechnology and BioMedicine, Shenzhen Key Laboratory of Gene & Antibody Therapy, State Key Laboratory of Health Science & Technology (prep) and Division of Life & Health Sciences, Graduate School at Shenzhen, Tsinghua University, Shenzhen, China; 4 School of Life Science, Tsinghua University, Beijing, 100084 China; 5 Key Laboratory of National Forestry and Grassland Administration for Orchid Conservation and Utilization at College of Landscape Architecture, College of Landscape Architecture, Fujian Agriculture and Forestry University, Fuzhou, China; 6 Fujian Colleges and Universities Engineering Research Institute of Conservation and Utilization of Natural Bioresources, College of Forestry, Fujian Agriculture and Forestry University, Fuzhou, 350002 China; 7 Orchid Research and Development Center, National Cheng Kung University, Tainan, 701 Taiwan; 8 Guangdong Key Laboratory of Ornamental Plant Germplasm Innovation and Utilization, Environmental Horticulture Research Institute, Guangdong Academy of Agricultural Sciences, Guangzhou, 510640 China; 9 Fujian Colleges and Universities Engineering Research Institute of Conservation and Utilization of Natural Biosciences, College of Forestry, Fujian Agriculture and Forestry University, Fuzhou, 350002 China

**Keywords:** Developmental biology, Gene expression analysis, Plant sciences

## Abstract

The ovules and egg cells are well developed to be fertilized at anthesis in many flowering plants. However, ovule development is triggered by pollination in most orchids. In this study, we characterized the function of a B_sister_ gene, named *PeMADS28*, isolated from *Phalaenopsis equestris*, the genome-sequenced orchid. Spatial and temporal expression analysis showed *PeMADS28* predominantly expressed in ovules between 32 and 48 days after pollination, which synchronizes with integument development. Subcellular localization and protein–protein interaction analyses revealed that PeMADS28 could form a homodimer as well as heterodimers with D-class and E-class MADS-box proteins. In addition, ectopic expression of *PeMADS28* in *Arabidopsis thaliana* induced small curled rosette leaves, short silique length and few seeds, similar to that with overexpression of other species’ B_sister_ genes in *Arabidopsis*. Furthermore, complementation test revealed that *PeMADS28* could rescue the phenotype of the *ABS/TT16* mutant. Together, these results indicate the conserved function of B_sister_ *PeMADS28* associated with ovule integument development in orchid.

## Introduction

In plants, MADS-box genes control the development of distinct organs, such as flower, ovule, fruit, leaf, and root^1–3^. Plant MADS-box genes can be classified into types I and II genes on the basis of phylogenetic analysis^4^. The best studied plant type II MADS-box transcription factors are those involved in floral organ identity determination. The determination of floral organ primordia by genes of the A, B, C, D and E classes led to the ABCDE model^5–10^. Furthermore, most of plant type II MADS-box proteins share a conserved structure consisting of four domains: MADS (M), intervening (I), keratin-like (K), and C-terminal (C)^11,12^. The DNA binding partner specificity is mediated to a large extent by the I domain, and the K domain likely promotes protein dimerization as well as tetramerization^13–15^. Proteins of floral MADS-box genes participating in floral organ identity interact with each other to control downstream genes^16–19^. Most of the protein–protein interactions necessary for the constitution of quaternary complexes, as recommended by the “quartet model”, are conserved^20^. Proliferating ovule primordia is specified by specific ovule identity factors, such as the MADS-box family members *SEEDSTICK* (*STK*), *SHATTERPROOF1* (*SHP1*), *SHP2*, *SEPALLATA* (SEP) and *AGAMOUS*^21–23^. Moreover, B_sister_ genes are “marker genes” for the development of (inner) integument structures, phylogenetically the “oldest” structures surrounding the female gametophyte of seed plants^24^.

In dicotyledons, the first B_sister_ gene was isolated from *Arabidopsis thaliana* and was named *ARABIDOPSIS BSISTER* (*ABS/TT16*)^25,26^. *ABS/TT16* is expressed mainly in the innermost integument layer, the endothelium; a closely related paralog is *GORDITA* (*GOA*, formerly known as *AGL63*). Besides study of eudicots, B_sister_ MADS-box genes have been investigated in the monocot *Oryza sativa*. *ABS/TT16* and *GOA* were found not functionally redundant in ovule development^24,26–28^. *ABS/TT16* is required for the proper differentiation of the inner integument, and *GOA* is necessary for at least the early development of the outer integument^28^. The single mutants *abs/tt16* and *goa* still produce seeds, which germinate properly^24,26,27^. In addition, silencing of *OsMADS29*, a B_sister_ gene in rice, led to severe phenotypes with degeneration of the pericarp, ovular vascular trace, integuments, nucellar epidermis and nucellar projection^29^.

Orchids, constituting approximately 10% of all seed plant species, have enormous value for commercial horticulture and are of specific scientific interest because of their extraordinary diversity of floral morphology, ecological adaptations, and unique reproductive strategies^30^. The unique reproductive strategies include mature pollen grains packaged as pollinia, pollination-regulated ovary/ovule development, synchronized timing of micro- and mega-gametogenesis for effective fertilization, and release of thousands or millions of immature embryos (seeds without endosperm) in mature pods^31^.

In most flowering plants, the ovules are mature, and the egg cells are ready for fertilization at anthesis. In contrast, in orchids, ovule development is triggered by pollination. In most orchids such as *Cattleya*, *Sophronitis*, *Epidendron*, *Laelia*, *Phalaenopsis*, *Dendrobium* and *Doritis*, ovules are completely absent in unpollinated ovaries, and the development of ovule is triggered only after pollination^32^. The long-term progressive process of ovule development in orchids as compared with other flowering plants is an attractive system for investigating ovule initiation and subsequent development.

With high economic value, *Phalaenopsis* orchids are beautiful ornamental plants and very popular worldwide. The genome of *P. equestris* was recently sequenced^33^ and the information provides a great opportunity to identify and characterize the genes involved in regulating orchid ovule development^34^. In this study, we identified and functionally characterized *PeMADS28*, a B_sister_ MADS-box gene, in *P. equestris*. Our results indicate that the function of *PeMADS28* plays an important role in ovule integument development in orchid and reveals the functional conservation of B_sister_ genes between monocots and dicots.

## Results

### Identification of *PeMADS28 *MADS-box gene in *P. equestris*

Only one B_sister_ MADS-box gene, *PeMADS28* (predicted proteome gene ID Peq004141), exists in the *P. equestris* genome^33^. The sequence of *PeMADS28* was retrieved from OrchidBase^35,36^. The ORF including 723 bp encodes a protein of 240 amino acids. Multiple sequence alignment with other B_sister_ proteins from gymnosperm, dicots and monocots demonstrated that PeMADS28 has a typical MIKC-type domain structure (Supplementary Fig. S1). B_sister_ proteins also contain a conserved PI Motif-Derived sequence in their C-terminal regions that are also representative of B-class MADS-box proteins (Supplementary Fig. S1)^25^.

### Phylogenetic relationship of PeMADS28 and other MADS-box genes

**Figure 1:**
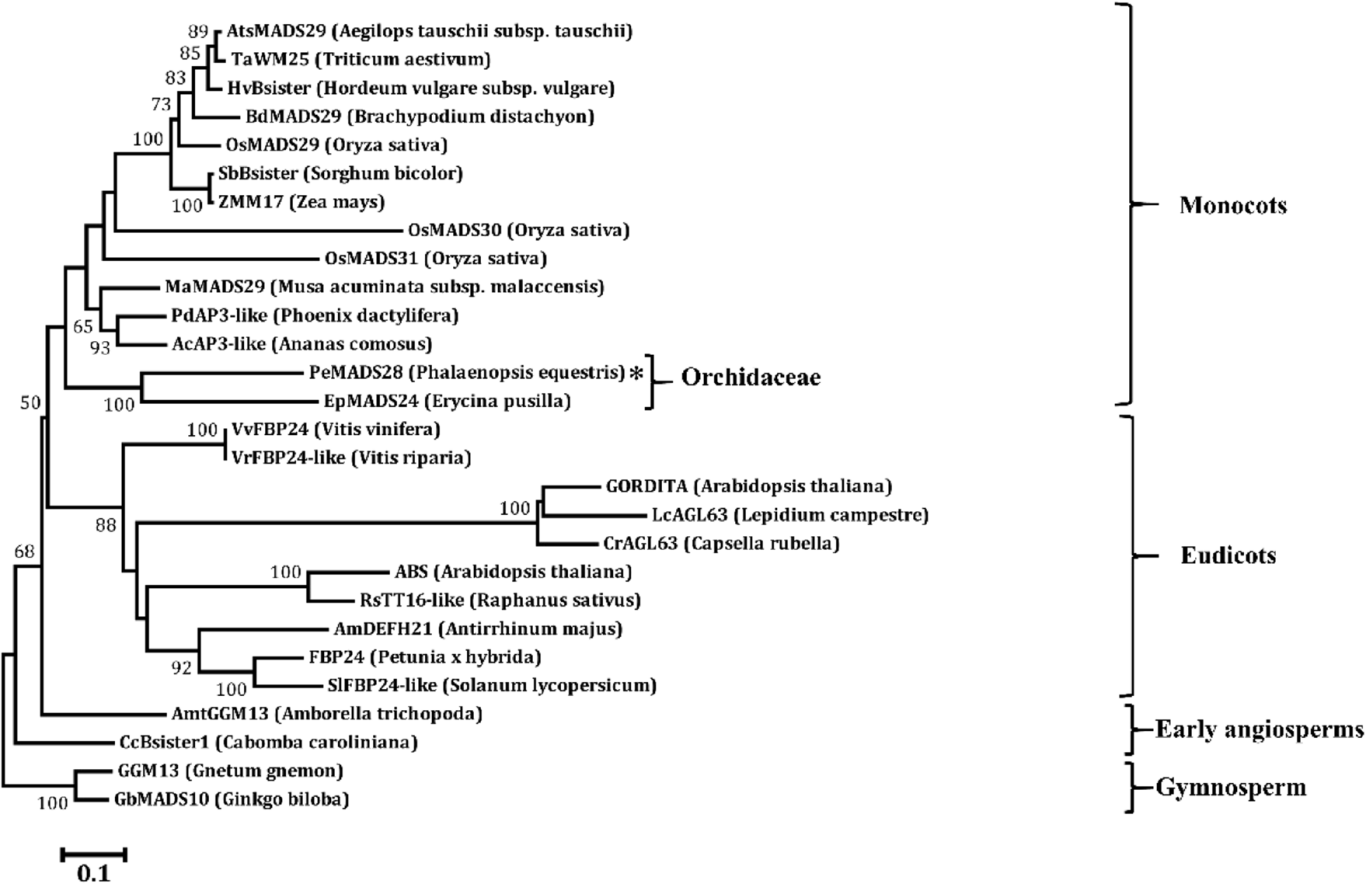
Phylogenetic analysis of B_sister_ proteins. GGM13 from *Gnetum gnemon* and GbMADS10 from *Ginkgo biloba* were outgroup representatives. Bootstrap values from 1000 replicates are indicated on most major nodes. PeMADS28 is highlighted by the asterisks.

To determine the phylogenetic relationships of PeMADS28 and other B_sister_ genes, we constructed a phylogenetic tree by using the amino acid sequences of PeMADS28 with other known gymnosperm and angiosperm B_sister_ sequences and the AGL63-like sequence from Brassicaceae. Amino acid sequences of B_sister_ proteins and AGL63-like proteins were retrieved from the National Center for Biotechnology Information (NCBI). This phylogeny has two supported major clades, one containing monocot B_sister_ proteins and the other dicot B_sister_ proteins (Fig. 1). Moreover, the B_sister_ and AGL63-like proteins were divided into two groups in dicots clade. PeMADS28 is close to the orchid *Erycina pusilla* B_sister_ protein EpMADS24 (Fig. 1). These results strongly suggest that *PeMADS28* belongs to the B_sister_ gene family.

### Spatial and temporal expression of *PeMADS28* in *P. equestris*

**Figure 2:**
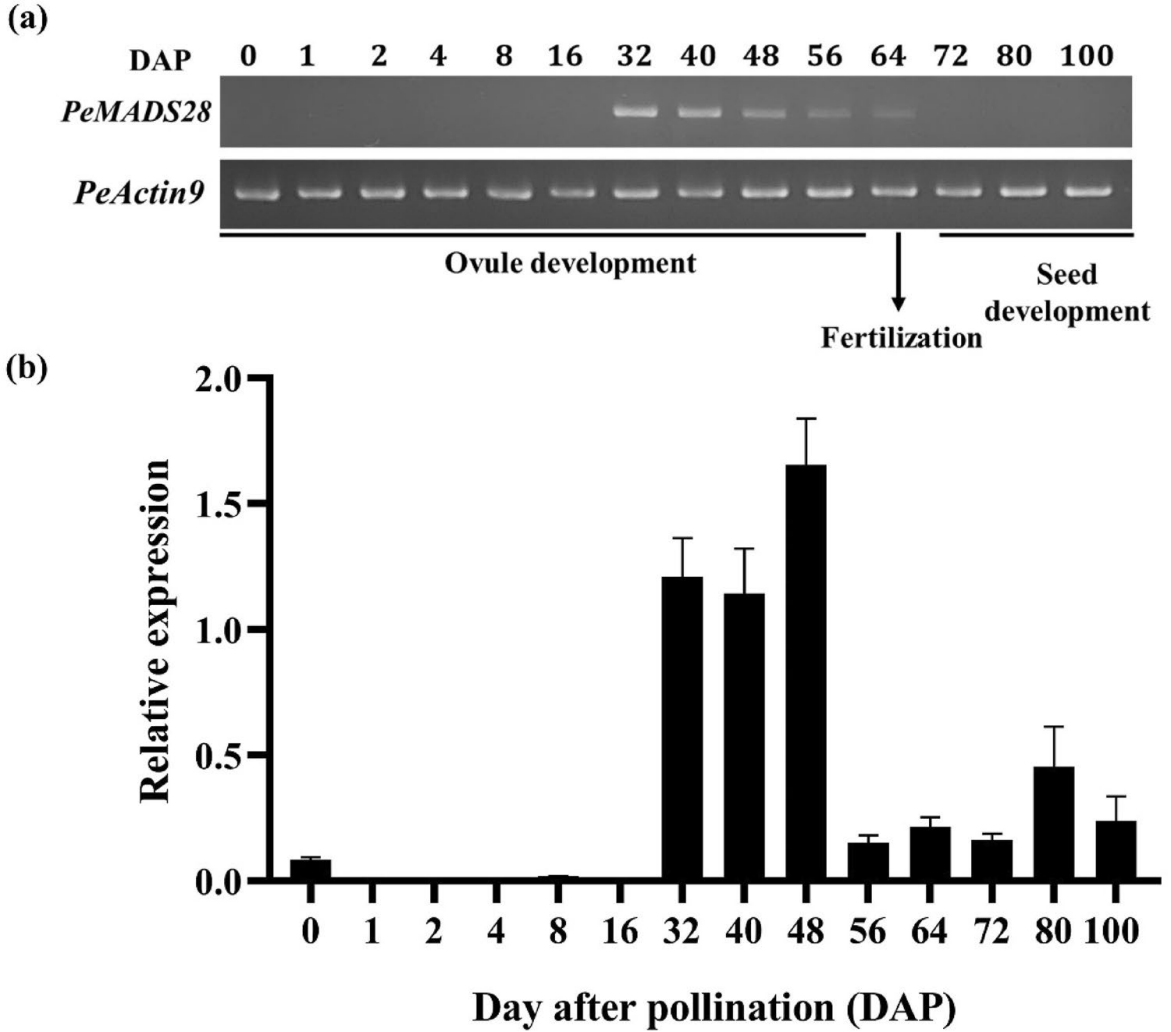
Expression patterns of *PeMADS28* at various developing ovule stages in *Phalaenopsis equestris* (**a**) RT-PCR analysis of *PeMADS28.* Expression of *Phalaenopsis actin* was an internal control. (**b**) Quantitative real-time RT-PCR analysis of *PeMADS28.* DAP: days after pollination.

RT-PCR and quantitative real-time RT-PCR were used to survey the spatial and temporal expression patterns of *PeMADS28*. Because B_sister_ MADS-box genes are involved in ovule development and pollination is a key regulatory event in orchid ovule initiation, we determined the temporal mRNA expression patterns of *PeMADS28* in developing ovules triggered by pollination. During ovule development, *PeMADS28* transcript level was highest from 32 to 48 days after pollination (DAP) (Fig. 2a,b), then decreased from 56 to 100 DAP (Fig. 2a,b). However, *PeMADS28* expression was barely observed in flower buds and was absent from vegetative tissues (Supplementary Fig. S2). Previous research showed that ovule development between 32 and 48 DAP is associated with inner and outer integument development^37^. These results suggest *PeMADS28* has functions in ovule integument development.

### In situ hybridization of *PeMADS28* transcripts

**Figure 3:**
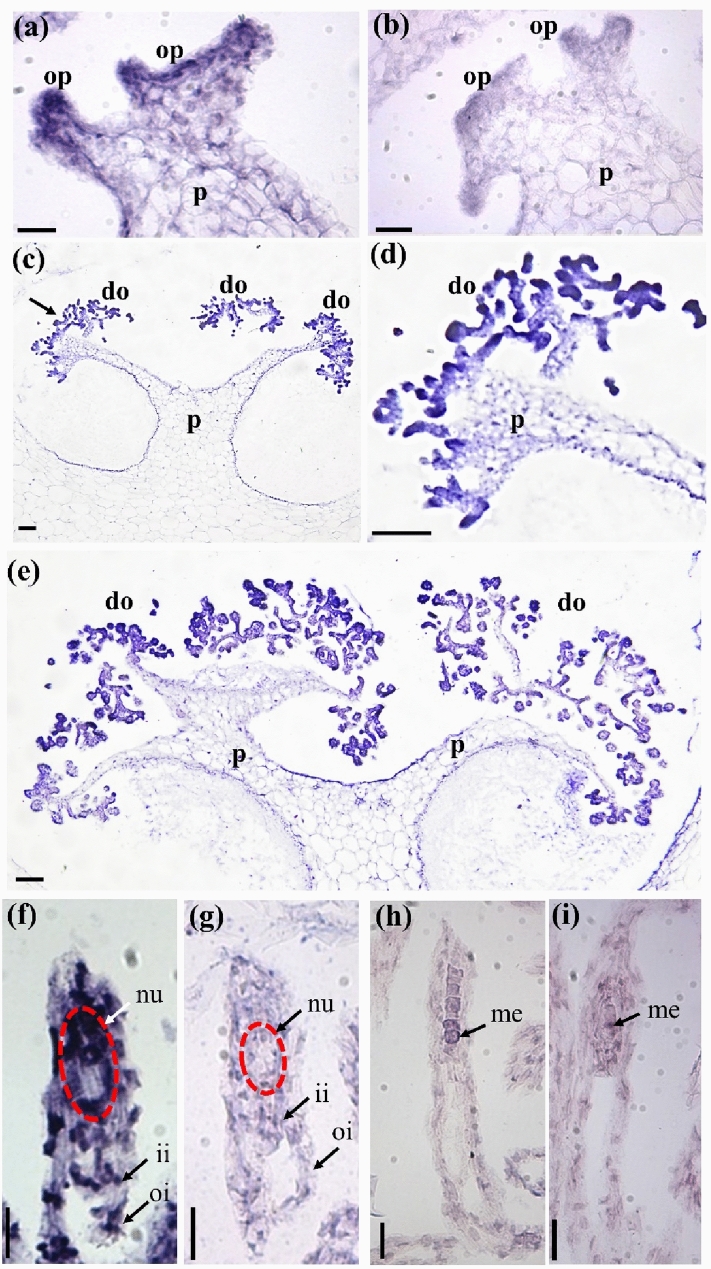
In situ hybridization of *PeMADS28* in developing ovules of *P. equestris*. (**a**,**b**) Placenta with ovule primordium at 4 DAP; (**c**) placenta with developing ovule at 32 DAP; (**d**) enlarged region of the dark arrow in (**c**); (**e**) placenta with developing ovule at 40 DAP; (**f**,**g**) developing ovules at 48 DAP; (**h**,**i**) developing ovule at 56 DAP. In (**a**), (**c**), (**d**), (**e**), (**f**) and (**h**), antisense probes were used to detect *PeMADS28* transcripts. In (**b**), (**g**) and (**i**), hybridization involved sense probes (negative controls). Bars, 0.1 mm. p, placenta; op, ovule primordium; do, developing ovule; ii, inner integument; oi, outer integument; nu, nucellus; me, megaspores; DAP: days after pollination. The nucellus is highlighted by the red dash line.

We further examined the detailed spatial and temporal expression patterns of *PeMADS28* during ovule development by in situ hybridization with antisense RNA probes. During the early stage of ovule development, when the final branches of placental protuberances differentiate ovular primordia, *PeMADS28* transcript expression was detected in all ovule primordia at their initiation (Fig. 3a,c,d). In the later stage, the expression was more concentrated in developing ovules (Fig. 3e). At 48 DAP, *PeMADS28* mRNA was detected in the whole ovule including nucellus and integument (Fig. 3f). *PeMADS28* transcript expression was not detected in 56-DAP ovules (Fig. 3h). The negative control was sense RNA used as a probe (Fig. 3b,g,i). These results supported that *PeMADS28* might be involved in orchid ovule initiation and integument development.

### Subcellular localization of PeMADS28-GFP fusion protein

**Figure 4:**
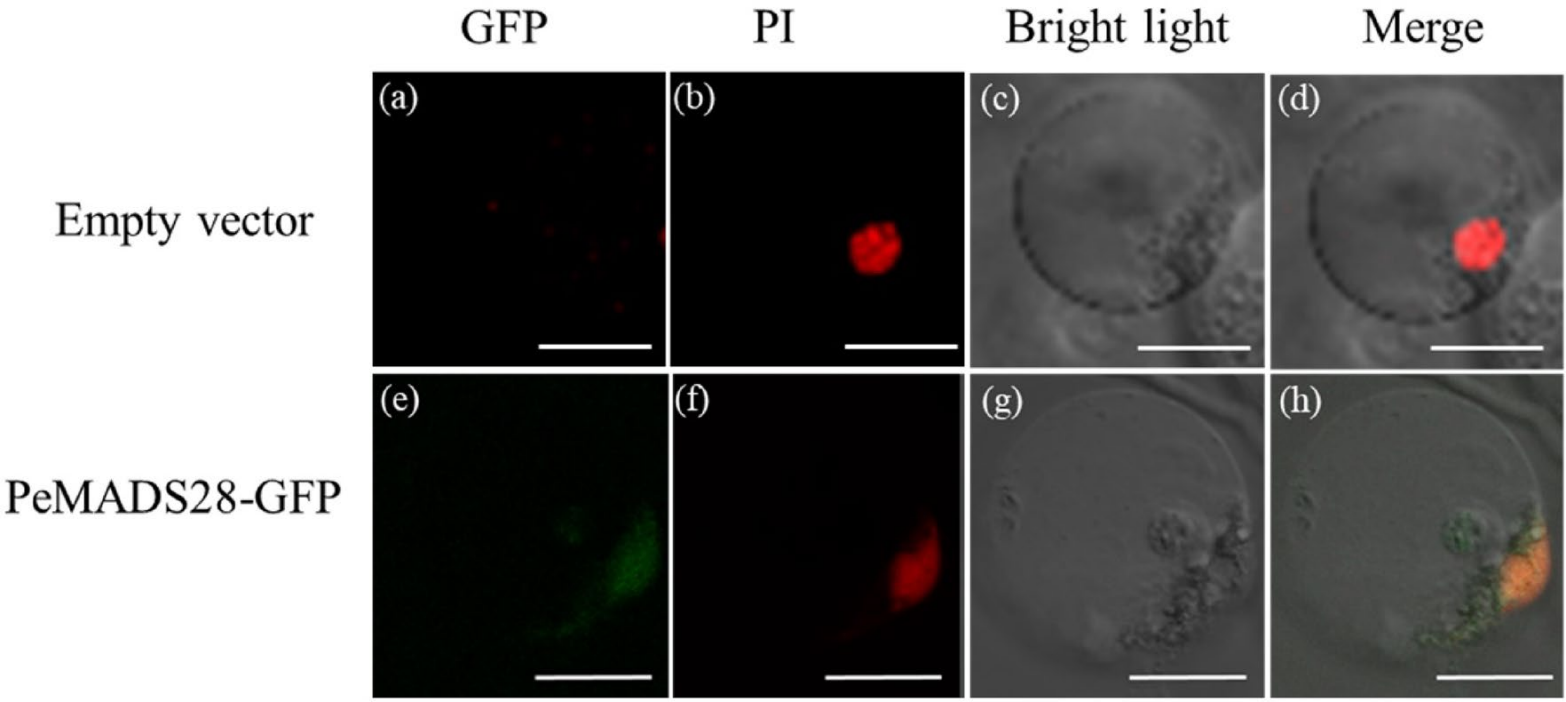
Localization patterns of PeMADS28-GFP fusions in *Phalaenopsis* protoplasts. Images show fluorescence and bright-field confocal microscopy images and merged images of flower protoplast. (**a**) Empty vector was no green fluorescence in the cytoplasm and nucleus. (**b**) Cell in (**a**) stained with propidium iodide (PI) represented in red to confirm the nucleus. (**c**) Cell in (**a**) and (**b**) by bright-field confocal microscopy. (**d**) Merged image of (**a**), (**b**) and (**c**) to confirm green fluorescence in the cytoplasm of a flower cell. (**e**) PeMADS28-GFP green fluorescence in nucleus and cytoplasm. (**f**) Cell in (**e**) stained with PI represented in red to confirm the nucleus. (**g**) Cell in (**e**) and (**f**) by bright-field confocal microscopy. (**h**) Merged image of (**e**), (**f**) and (**g**) to confirm green fluorescence in cytoplasm. Bars: 20 µm.

As a member of MADS-box transcription factors, PeMADS28 was expected to localize in the nucleus. To test the subcellular localization of PeMADS28, a PeMADS28-GFP fusion protein was generated with a GFP reporter gene fused in-frame to the PeMADS28 coding region under control of the 35S promoter. Transient expression of PeMADS28-GFP fusion protein was analyzed in *Phalaenopsis* petal protoplasts. PeMADS28-GFP fusion protein signals were observed in both the nucleus and cytoplasm along with GFP signals (Fig. 4). Thus, PeMADS28 might need to interact with other proteins to exclusively localize in the nucleus.

### Interaction behavior of PeMADS28 analyzed by bimolecular fluorescence complementation (BiFC) assay

**Figure 5:**
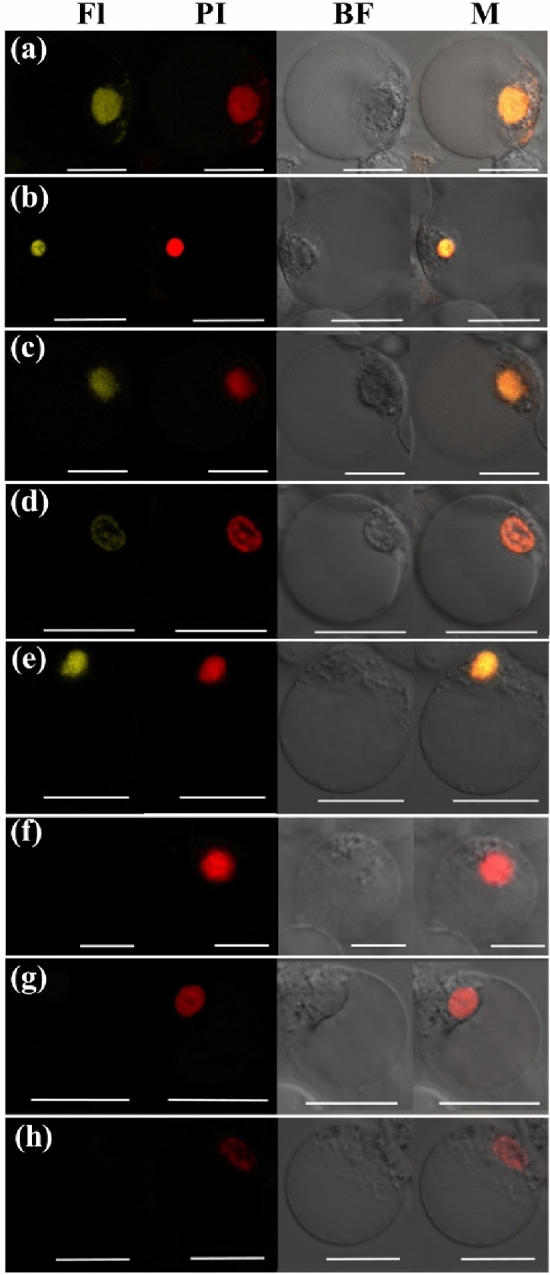
Analysis of protein–protein interactions among B_sister_ PeMADS28, C-class PeMADS1, D-class PeMADS7 and E-class PeSEP3 proteins by BiFC method. Fusion proteins were expressed in *Phlaenopsis* petal protoplasts. (**a**) PeMADS28:YFPc + PeMADS28:YFPn. (**b**) PeMADS28:YFPc + PeSEP3:YFPn. (**c**) PeSEP3:YFPc + PeMADS28:YFPn. (**d**) PeMADS28:YFPc + PeMADS7:YFPn. (**e**) PeMADS7:YFPc + PeMADS28:YFPn. (**f**) PeMADS28:YFPc + PeMADS1:YFPn. (**g**) PeMADS1:YFPc + PeMADS28:YFPn. (**h**) YFPc + YFPn as a negative control. BF, bright field; Fl, fluorescence image; M, Merged image; PI, propidium iodide. Bars, 20 µm.

A number of previous studies demonstrated that MADS-box transcription factors form dimers or higher-order complexes for their functions in flower and ovule development^17,20,21,24,38–40^. To investigate the ability of homodimer formation of PeMADS28 and nuclear localization of this self-association, we used BiFC assay. A BiFC vector pair with PeMADS28 fused to N- or C-terminal halves of YFP (PeMADS28:YFPn and PeMADS28:YFPc) was prepared and used to co-transfect *Phalaenopsis* petal protoplasts. Fluorescence YFP signal clearly indicated an interaction between the two PeMADS28 monomers. The formed homodimer was exclusively localized in the nucleus (Fig. 5a). These results demonstrate that dimerization of PeMADS28 monomers plays an important role in retaining PeMADS28 in the nucleus. Previous reports indicated that PeMADS1 (C-class), PeMADS7 (D-class), and PeSEP3 (E-class) MADS-box genes are involved in orchid ovule development^37,41^. To gain more insight into the interaction of MADS-box proteins involved in orchid ovule development, interaction behaviors among Bs and C-class, D-class, and E-class proteins were further investigated by BiFC assay. Interaction fluorescence signals were observed in the combination of PeMADS28 and PeSEP3 (Fig. 5b,c) as well as PeMADS28 and PeMADS7 (Fig. 5d,e), which suggests that the orchid B_sister_ protein can interact with E- and D-class MADS-box proteins. In addition, the signals were localized in the nucleus, as indicated by use of the nuclear dye propidium iodide (PI). However, interaction was not observed with the combination of PeMADS28 and PeMADS1 (Fig. 5f,g). Therefore, PeMADS28 may not form heterodimers with C-class MADS-box proteins. No fluorescence was detected with the empty vector control (Fig. 5h).

### Functional analysis of *PeMADS28 *gene by ectopic expression and complementation in *Arabidopsis thaliana*

**Figure 6:**
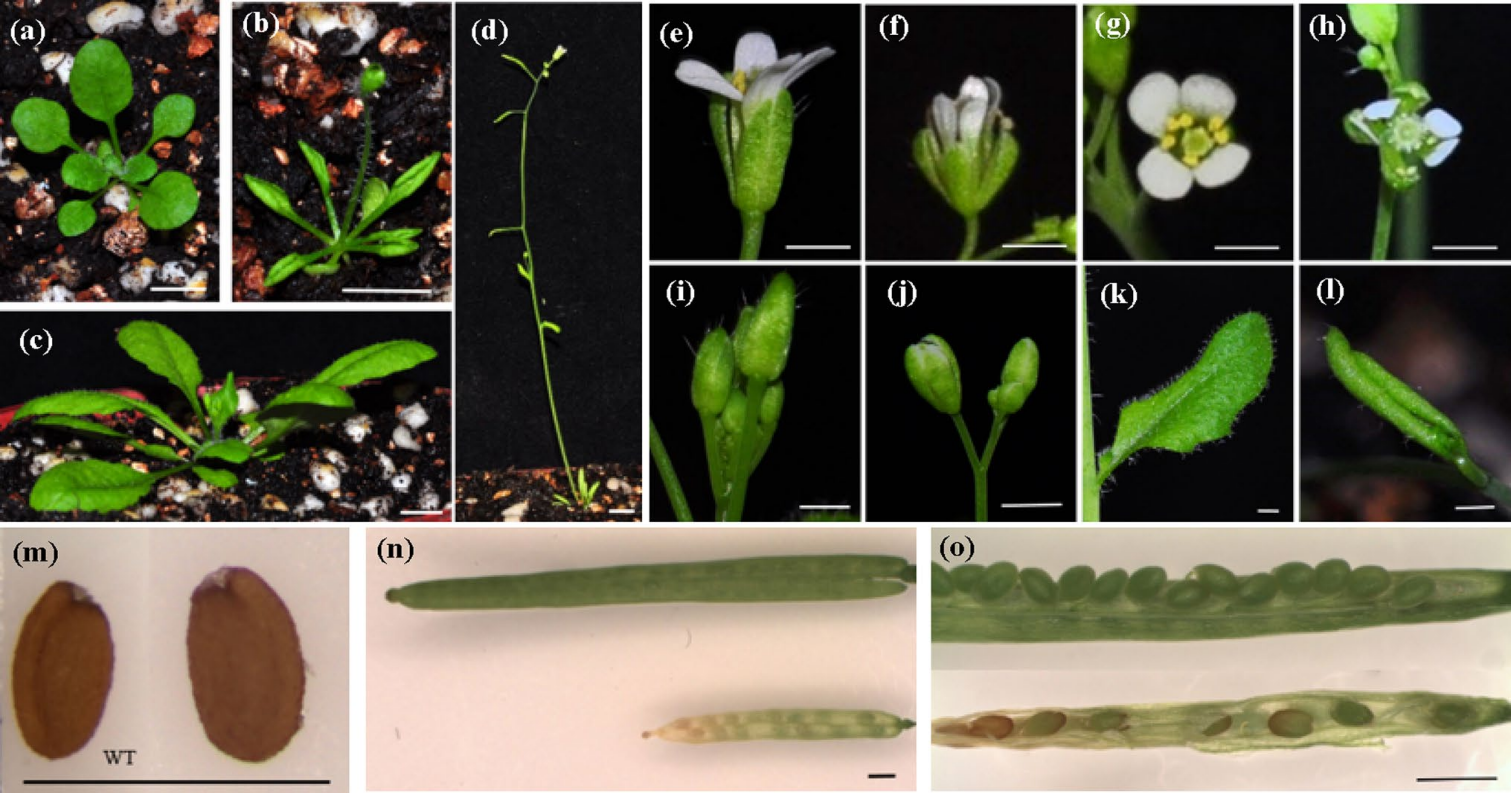
Phenotype analysis of transgenic *Arabidopsis* overexpressing *PeMADS28*. (**a**) 20-day-old wild-type plant; (**b**) 20-day-old *35S::PeMADS28* transgenic plant; (**c**) 31-day-old wild-type plant; (**d**) 31-day-old *35S::PeMADS28* transgenic plant. Bars (**a**–**d**), 5 mm. (**e**) Side-view of wild-type flower; (**f**) side-view of *35S::PeMADS28* transgenic flower; (**g**) top-view of wild-type flower; (**h**) top-view of *35S::PeMADS28* transgenic flower; (**i**) Wild-type floral inflorescence; (**j**) *35S::PeMADS28* transgenic floral inflorescence; (**k**) cauline leaf of wild-type plant; (**l**) cauline leaf of *35S::PeMADS28* transgenic plant. Bars (**e**–**l**) 1 mm. (**m**) Wild-type seed (left); *35S::PeMADS28* transgenic seed (right); (**n**) silique of wild-type (upper); silique of *35S::PeMADS28* transgenic plant (lower); (**o**) silique of wild-type without one valve (upper); silique of *35S::PeMADS28* transgenic plant without one valve (lower). Bars (**m**–**o**) 1 mm.

For functional characterization of *PeMADS28*, we constructed transgenic *Arabidopsis* plants expressing *PeMADS28* under control of the cauliflower mosaic virus (CaMV) 35S promoter via *Agrobacterium*-mediated transformation. A total of 20 independent overexpressed *PeMADS28* transgenic lines were obtained based on kanamycin selection and a similar phenotype. Among twenty transgenic lines, nine showed a 3:1 segregating kanamycin resistance phenotype. As compared with wild-type plants, six independent *PeMADS28* overexpressed lines shows the early flowering phenotype (Fig. 6a,c [wild-type plant]; 6b,d [transgenic plant]) and fewer flower bud production (Fig. 6i [wild-type plant]; 6j [transgenic plant]). The rosette and cauline leaves of transgenic plants had upwardly curled profiles and were smaller than those of wild-type plants (Fig. 6a,k [wild type plant]; 6b,l [transgenic plant]). No homeotic conversion of floral organs was observed in transgenic plants. Moreover, transgenic plants had smaller flowers with cracked sepals than wild-type plants (Fig. 6e,g [wild-type plant]; 6f,h [transgenic plant]). The length of siliques was shorter in transgenic plants (Fig. 6n and Table 1). Transgenic plants also showed more undeveloped seeds in siliques than did wild-type plants (Fig. 6o and Table 1). In addition, transgenic seeds were larger and heavier (Fig. 6m and Table 1). Previously, *GORDITA* and *ABS*/*TT16* are the paralogs in *Arabidopsis*. Consistently, over-expressed the *GORDITA* or *ABS*/*TT16* in *Arabidopsis* caused that the plant size shorter, and all organs are smaller than those in the wild-type^24,26,28^. Both two over-expressed plants were affected the fruit development, and *ABS*/*TT16* led to the rosette leaves curled^24,26,28^. It is similar to *35::PeMADS28* phenotype. These results appeared that the *PeMADS28* may play a role in fruit development.

**Table 1:** Silique length and seeds in OX*PeMADS28* transgenic plants and wild-type (WT) plants.

Plant	WT	OX*PeMADS28*
Silique length (cm)	1.29 ± 0.13 (n = 15)	0.85 ± 0.16 (n = 15), decrease*
Seeds/silique	45.33 ± 3.81 (n = 15)	24.26 ± 4.04 (n = 15), decrease*
100 seeds (mg)	3.04 ± 0.4 (n = 3)	5.05 ± 0.2( n = 3), increase*

Asterisks indicate statistically significant differences (*P < 0.05 compared with WT by Student’s t-test); The ± standard deviation (SD) of the three biological repeats.

**Figure 7:**
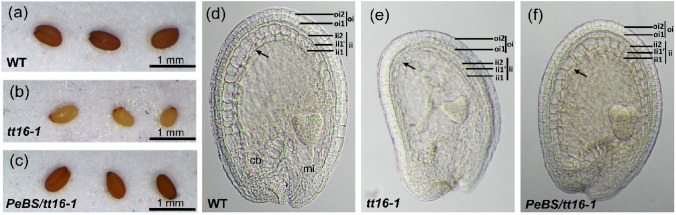
Phenotypes of seed pigmentation and structure of the seed coat. Seed pigmentation of mature seeds from wild-type (Col) (**a**), a *tt16-1* mutant (**b**), and transgenic *35S::PeMADS28* in *tt16-1* mutant plant (**c**). The development of seed coat from cleaned seed in wild-type (Col) (**a**), *tt16-1* mutant (**b**), and transgenic *35S::PeMADS28* in *tt16-1* mutant plant (**c**). The dark arrows are indicated the endothelium. cb, chalazal bulb; ii, inner integument; mi, micropyle; oi, outer integument.

To further validate the function of *PeMADS28*, we used complementation testing with the *tt16-1* mutant and examined the seed pigmentation and development of the endothelium. A total of 8 transgenic lines were obtained and 4 showed a 3:1 segregating kanamycin-resistance phenotype. All of the T2 line seeds showed restoration of pigmentation to a brown color from the straw color of the *tt16-1* mutant (Fig. 7a–c). In addition, *PeMADS28* could rescue the development of endothelium in *tt16-1* plants. Endothelial cells in immature wild-type seeds were small, almost rectangular in shape and regularly spaced (Fig. 7d–f). In *abs*/*tt16* immature seeds, endothelium cells seemed to be flatter and more irregularly shaped than wild-type cells, resembled parenchymatic cells, and often seemed to collapse (Fig. 7f)^26^. All of the T2 line (*35S::PeMADS28* transgenic *tt16-1*) seed coats showed the normal endothelium of the wild-type seed coat (Fig. 7e), so *PeMADS28* was sufficient to complete the function of *Arabidopsis ABS*/*TT16*.

## Discussion

In this work, we identified a B_sister_-like gene, *PeMADS28*, from the *P. equestris* genome and characterized its function by sequence comparison, expression profile analysis, protein–protein interaction behavior, ectopic expression and complementation experiments in *Arabidopsis*. Protein sequence alignment showed that PeMADS28 is a typical B_sister_ protein with respect to its protein sequence because it contains a conserved sub-terminal “PI motif-derived sequence,” which is also representative of B-class MADS-box proteins^25^. Phylogenetic analysis with use of a deduced amino acid sequence revealed that *PeMADS28* belongs to the monocot *B*_*sister*_ subclade. Both analyses suggested that *PeMADS28* is a putative orchid ortholog of B_sister_ genes like *ABS/TT16* from *Arabidopsis*.

B_sister_ genes are closely related to B-class genes but express predominantly in the female reproductive organ. Previous studies showed that B_sister_ genes are expressed in the ovule and envelope in gymnosperms and in the ovule and integuments of angiosperms. In the gymnosperm *Gnetum gnemon*, expression of B_sister_ *GGM13* is specifically strong at the adaxial base of the cupules, where ovules subsequently develop^42^. When ovules appear, *GGM13* expression is limited to the developing nucellus and inner envelopes^42^. In dicots, the *Arabidopsis* B_sister_ gene *ABS/TT16* is expressed mainly in endothelium^43^. In petunia, *FBP24* is expressed in young ovule primordia, nucellus and integument. Later, the expression is confined to the endothelium in mature ovules^38^. In snapdragon, *DEFH21* expression was found in only a few inner cell layers of the inner integuments of the ovules^25^. In monocots, wheat *WBsis* mRNA was detected in the developing inner integument at the late floral organ developmental stage^44^. In rice, *OsMADS29* transcripts are localized in the ovule, including integuments and nucellus throughout ovule development^29^.

These results reveal a similarity of expression of B_sister_ genes suggesting conservation of the gene expression pattern over at least 300 million years^27^. In our study, temporal expression analysis revealed significant *PeMADS28* transcript expression between 32 and 48 DAP (Fig. 2a,b). In addition, in situ hybridization signals of *PeMADS28* transcripts were concentrated in the developing ovules (Fig. 3c–f). Hence, B_sister_ genes may have conserved expression patterns in seed plants. Interestingly, although *Arabidopsis* genome contains two and rice has three B_sister_ genes, these homologous genes have been occurred diversified expression and functional differentiation. *ABS/TT16* is involvement in endothelial cell specification and control of flavonoid biosynthesis in *Arabidopsis* seed coat^26^. The *GOA* is a young paralog of *ABS/TT16* and play a role in fruit longitudinal growth^27^. The rice *OsMADS29* was identified as a key regulator of early rice seed development by regulating the programmed cell death of maternal tissues^29^. *OsMADS30* does not have a canonical ‘B_sister_ function’, and revealed neo-function in shoot size and architecture^45^. In fact, the development of orchid ovule is the typical monosporic Polygonum type in which the functional megaspore passes through three mitotic divisions producing a seven celled embryo sac consisting of three antipodal cells, one central cell formed by two polar nuclei, two synergid cells, and the egg cell. In addition, the embryo sac is enclosed by inner and outer integuments. The orchid ovule structure and development is highly similar to that of *Arabidopsis* and cereal except that the inner integument gradually degenerated during the early stages of embryo proper formation and ovule initiation and development is precisely triggered by pollination. Our data considered that the B_sister_ gene *PeMADS28* might involve in the typical ovule development including integument morphogenesis.

Previously, it has been shown that the antagonistic development of nucellus and endosperm in *Arabidopsis*^46^. The endosperm delivers the signal for the differentiation of seed coat and then both of tissues orchestrates seed growth. However, the endosperm could also initiate nucellus degeneration via vacuolar cell death and necrosis^46^. It also has been demonstrated that TT16/ABS can regulate proanthocyanidins synthesis in the seed coat and conversely *TT16/ABS* expression in the seed coat is sufficient to activate the nucellus degeneration^46^. In *Phalaenopsis* orchids, double fertilization could be observed. However, the triple fusion nucleus of the endosperm initial is amorphous in shape and apparently begins to degenerate immediately, consequently forming no endosperm^47,48^. We speculated that the signal generated by fertilization of the central cell triggers its degeneration through activation of the *PeMADS28* expression. Because endosperm initial lives shortly, the signal might not spread to the seed coat. In fact, the *Phalaenopsis* seed coat do not accumulate proanthocyanidins^49^. However, whether signal generated by fertilization of the central cell could reach to the nucellus and initiates the nucellus cell death should be necessary for further study.

As transcriptional regulatory proteins, a number of MADS-box proteins have been shown to localize in the nucleus. However, some MADS-box proteins are unable to translocate into the nucleus by themselves, but their dimers deposit in the nucleus; examples are AP3-PI^50^ and UNSHAVEN-FLORAL BINDING PROTEIN 9 (FBP9)^51^. In this study, we detected PeMADS28-GFP fusion proteins in the nucleus and cytoplasm (Fig. 4h). However, BiFC results showed the PeMADS28 homodimer specifically retained in the nucleus. The results suggest that homodimerization of PeMADS28 drives a conformational change to bring it into a nuclear-retaining structure. This kind of behavior of orchid B_sister_ PeMADS28 is similar to that of rice OsMADS29^52^. Our data suggest the probability of PeMADS28 being regulated at the post-translational level via its interactions, which may affect its function by regulating entry into the nucleus and regulation of its targets.

In *Arabidopsis*, previous study suggested a specific interaction of ABS with STK, SEP3, SHP1 and (much weaker) SHP2 but not AG^24,38^. OsMADS29 could interact with OsMADS3 (C-class proteins) and all five E-class proteins of rice^52^. Our results indicate that PeMADS28 can form a homodimer in the nucleus. In addition, it could interact with D-class (PeMADS7) and E-class (PeMADS8) MADS-box proteins. However, PeMADS28 and PeMADS1 may not form heterodimers directly. These results suggest that protein interaction behaviors among B_sister_, D- and E-class proteins are conserved in angiosperms. Furthermore, in previous study, protein–protein interaction analyses revealed that PeSEP3 could bridge the interaction between PeMADS1 and PeMADS7 involved in *Phalaenopsis* gynostemium and ovule development^37^. Thus, a higher-order protein complex formed by C-E-D-B_sister_ genes (PeMADS1-PeMADS8-PeMADS-PeMADS28) might have an important role in regulation of orchid ovule development.

Functional analysis of *ABS* has shown abnormal characteristics in vegetative and reproductive organs of *ABS*-overexpressing *Arabidopsis*, including curled rosette leaves, late flowering, small flowers and shrunken siliques with few developed seeds^26^. Overexpression of *GOA*, the paralog of *ABS*, showed similar phenotypes as *ABS*-overexpressed plants, except that *GOA*-overexpressing plants displayed early flowering^27^. Overexpression of the *Ginkgo* B_sister_ gene *GBM10* in tobacco resulted in reduced size of transgenic seedlings, small and curled leaves, small flowers, small fruit with wrinkled surface and massive abortion of undeveloped ovules^42^. Similar to these phenotypes, our *PeMADS28*-overexpression *Arabidopsis* showed curled and small rosette leaves, early flowering, small flowers, short siliques and few developed seeds. Overexpressing *PeMADS28* in wild-type *Arabidopsis* demonstrated that *PeMADS28* has functions similar to those of B_sister_ genes in regulating ovule development. Moreover, overexpression of *PeMADS28* could restore the development of endothelial cells in the *tt16* mutant. Conserved functions of orchid B_sister_ genes for specifying integument development could occur in developing seeds of *Arabidopsis*, which indicates that a competent endothelium is needed for *PeMADS28* function to specify integument development.

In most orchids, ovary and ovule development is precisely triggered by pollination^32^. Previous studies showed that pollination inhibits *PeMADS6* (*B-PI* MADS-box gene) expression in the ovary via the auxin signaling pathway to promote *Phalaenopsis* ovary/ovule development^32,53^. In addition, expression of C-class *PeMADS1* and D-class *PeMADS7* was significantly induced by pollination^37^. Furthermore, the TCP gene *PeCIN8* showed a parallel expression pattern in the developing ovules of *Phalaenopsis* to that of *PeMADS28*^34^. Understanding the interaction as well as regulation networks of these genes, then stimulating pollination will help in further exploring the molecular mechanism of orchid ovule development. Moreover, the availability of several whole-genome sequences of orchids, including *P. equestris*, *Dendrobium catenatum*, and *Apostasia shenzhenica*^33,54–56^, can lead to promising exploration of more genes involved in the orchid ovule development.

## Materials and methods

### Plant materials and growth conditions

The plants of wild-type *P. equestris* (S82–159) were grown in greenhouses under natural light and controlled temperature from 23 to 27 °C^48^. *A. thaliana* ecotype Columbia was used in transformation experiments. Seeds were surface-sterilized in 10% (v/v) bleach for 15 min, then rinsed 3–4 times with sterile water. Sterilized seeds were grown on half-strength Murashige and Skoog medium (INVITROGEN, CARLSBAD CA, USA) in the presence of 1% (w/v) sucrose and 0.8% (w/v) agar. Plated seeds were incubated at 4 °C for 48 h, then maintained in a fully automated growth chamber (CHIN HSIN, Taiwan) under a 16-h light/8-h dark photoperiod at 22 °C for 10 days before being transplanted to soil^37^.

### Sequence alignment and phylogenetic analysis

Sequence alignment involved use of CLUSTALW and phylogenetic analysis MEGA 6 by the neighbor-joining method. Bootstrap analysis was with 1000 replicates. The GeneBank accession numbers for amino acid sequences are AtsMADS29 (XP_020188803), TaWM25 (CAM59071), HvBsister (BAK06913), BdMADS29 (NP_001288325), OsMADS29 (XP_015624837), SbBsister (XP_002453370), ZMM17 (NP_001105130), OsMADS30 (Q655V4), OsMADS31 (Q84NC2), MaMADS29 (XP_018678849), PdAP3-like (XP_00880798), AcAP3-like (XP_020109780), PeMADS28 (KT865880), EpMADS24 (AHM92100), VvFBP24 (RVW42148), VrFBP24-like (XP_034698718), ABS (Q8RYD9), RsTT16-like (XP_018481949), AmDEFH21 (CAC85225), FBP24 (AAK21255), SlFBP24-like (XP_019066630), AmtGGM13 (XP_006829168.2), CcBsister1 (ADD25185), GGM13 (CAB44459), GbMADS10 (BAD93174), GORDITA (NP_174399.2), CrAGL63 (XP_006306362.2), LcAGL63 (APB93359).

### RNA extraction

We collected unpollinated ovaries; ovaries and ovules at 1, 2, 4, 8 day after pollination (DAP); ovules at 16, 32, 40, 48, 56, 64 DAP; and developing seeds at 80 and 100 DAP from *P. equestris*^37^. Samples were immersed in liquid nitrogen, and stored at – 80 °C until the RNA was extracted. Total RNA was isolated with use of TRIZOL reagent (SIGMA-ALDRICH). Briefly, frozen tissue (0.5–1 g) was ground with liquid nitrogen with a pestle and mortar and homogenized in TRIZOL reagent. Then the dissolved RNA was extracted with chloroform. After centrifugation in 13,000 rpm to remove insoluble material, total RNA was precipitated with isopropanol and 0.8 M sodium citrate was added to dissolve polysaccharides at − 20 °C overnight; then samples were precipitated again with 4 M LiCl, pelleted, washed, and the final RNA precipitate was dissolved in a suitable volume of sterilized DEPC-treated water. Before cDNA synthesis, RNA was treated with RNase-free DNase I (INVITROGEN) to remove DNA contamination.

### RT-PCR and quantitative real-time PCR

RNA was used as a template for cDNA synthesis with reverse transcriptase and the SuperScript II kit (INVITROGEN). Transcripts of *PeMADS28* were detected by RT-PCR with gene-specific primers (Supplementary Table S1) for 25–30 cycles. The RT-PCR program was 95 °C for 7 min for denaturation of DNA and activation of polymerase, then amplification at 95 °C for 30 s, 55 °C for 30 s, 72 °C for 30 s and extension at 72 °C for 10 min as described previously^37^. The amplified products were analyzed on 1% agarose gels. Quantitative real-time PCR involved using the ABI Prism 7000 sequence detection system (APPLIED BIOSYSTEMS) with 2X SYBR green PCR master mix (APPLIED BIOSYSTEMS)^34^. Reaction involved incubation at 50 °C for 2 min, then 95 °C for 10 min, and thermal cycling for 40 cycles (95 °C for 15 s and 60 °C for 1 min). The relative quantification was calculated according to the manufacturer’s instructions (APPLIED BIOSYSTEMS)^34^. The expression of *PeActin4* (PACT4, AY134752) was used for normalization^34^. Primers used for amplification are in Supplementary Table S1.

### In situ hybridization

Developing ovules and developing seeds of *P. equestris* were fixed in 4% (v/v) paraformaldehyde and 0.5% (v/v) glutaraldehyde for 24 h at 4 °C, dehydrated through an ethanol series, embedded in Histoplast and longitudinal sectioned at 6–8 μm with use of a rotary microtome. Tissue sections were deparaffinized with xylene, rehydrated through an ethanol series, pre-treated with proteinase K (2 μg ml^−1^) in 1 × phosphate-buffered saline (PBS) at 37 °C for 60 min, acetylated with 0.5% acetic anhydride for 10 min, and dehydrated with an ethanol series. The resulting PCR fragments were used as templates for synthesis of both antisense and sense riboprobes with digoxigenin-labeled UTP-DIG (ROCHE APPLIED SCIENCE) and the T7/SP6 Riboprobe in vitro Transcription System (PROMEGA) following the manufacturer’s instructions. For quality control, hybridization probes were tested by using dot blot to analyze the sensitivity before in situ hybridization. Hybridization and immunological detection of signals with alkaline phosphatase were performed as described^48^.

### Subcellular localization of PeMADS28-GFP fusion protein

Template-specific primers were designed by the addition of an attB1 adapter primer (5′-GGG GAC AAG TTT GTA CAA AAA AGC AGG CTG G-3′) to the 5′ end of the first 18–25 nt of the open reading frame (ORF) and attB2 adapter primer (5′- GGG GAC CAC TTT GTA CAA GAA AGC TGG GTT-3′) to the 3′ end of the first 18–25 nt of the ORF, which generated the full-length attB1 and attB2 sites flanking the ORF (Supplementary Table S1). Gateway -compatible amplified ORFs were recombined into the pDONR 221 vector (INVITROGEN) by BP cloning: 1 µl (15–150 ng) PCR products, 2 µl BP clonase II Enzyme Mix (INVITROGEN), 150 ng pDONR vector plasmid and TE buffer (pH 8.0) were incubated at 25 °C for 1 h. Entry clones were used directly for transformation of *E. coli* DH5α cells, and bacteria were plated on LB medium containing 50 µg/ml of kanamycin. These entry clones were for recombination of target genes into the destination vector p2GWF7, C-terminal fusions^57^ in a reaction mixture containing 2 µl LR clonase II Enzyme Mix (INVITROGEN), 150 ng p2GWF7 vector, and TE buffer (pH 8.0), and incubation at 25 °C for 1 h. The LR reactions were used for transformation, then transformants were selected in plates containing 50 µg/ml ampicillin. The plasmids were transfected into *Phalaenopsis* protoplasts by PEG transformation. After culturing for 16 h, signals were visualized under a confocal laser microscope (CARL ZEISS LSM780, Instrument Development Center, NCKU). Separate bright field and fluorescence images were overlaid by using Axio Vision 4 Rel.4.8.

### Bimolecular fluorescence complementation assay (BiFC)

To construct the interaction vectors, we used gene-specific primers with an additional attB1 adapter primer (5′-GGG GAC AAG TTT GTA CAA AAA AGC AGG CTG G-3′) added to the 5′ end of the first 18–25 nt of the ORF and attB2 adapter primer (5′- GGG GAC CAC TTT GTA CAA GAA AGC TGG GTT-3′) to the 3′ end of the first 18–25 nt of the ORF by using Pfu DNA polymerase. Primers for amplification are in Supplementary Table S1. Gateway compatible amplified ORFs were recombined into the pDONR 221 vector (INVITROGEN) by BP cloning described previously^34^. Gateway LR clonase enzyme mix was used for cloning the entry clones into the BiFC destination vectors pSAT4-DEST-nEYFP-C1 (pE3136) and pSAT5(A)-DEST-cEYFP-N1 (pE3132). After the LR reactions, plasmids were transformed into DH5α cells and transfected into *Phalaenopsis* protoplasts by PEG transformation^34^. Signals were visualized by confocal laser microscopy (CARL ZEISS LSM780, Instrument Development Center, NCKU).

### *Arabidopsis* transformation

cDNA fragments containing the coding regions of *PeMADS28* were cloned into the pBI121 vector (primers are in Supplementary Table S1). Constructs were then introduced into *Agrobacterium tumefaciens* (strain GV3101). GV3101 was inoculated drop-by-drop into closed floral buds by using a micropipette. *Arabidopsis* transformation was modified by the addition of 0.05% (v/v) Silwet L-77 (LEHLE SEEDS, ROUND ROCK, TX, USA) in the transformation media. To select transformed *Arabidopsis*, seeds (T0) were screened on media supplemented with 50 μg/ml kanamycin (SIGMA- ALDRICH). After 2 weeks of selection, the kanamycin-resistant seedlings (T1) were transferred to soil and grown under the conditions described above. Kanamycin segregation in the T1 generation was analyzed by chi-square test. The homozygous, kanamycin-resistant T2 generation was used to confirm the integration fragment by PCR for each construct. Transformed lines with segregation ratio 3:1 were collected for further analysis. The seeds of *35S::PeMADS28* transgenic *Arabidopsis* plants were grown in the same environment as described previously^37^.

### Complementation assay

The *tt16-1* mutant was obtained from Dr. L. Lepiniec (Institut Jean-Pierre Bourgin, France^26^). The pBI121-PeMADS28 construct was transformed into the *tt16-1* mutant and screened on media supplemented with 50 μg/ml kanamycin. The seeds of *35S::PeMADS28 tt16-1* transgenic *Arabidopsis* plants were used.

### Differential interference contrast (DIC) microscopy

Immature seeds were removed from different developmental stages of siliques and soaked overnight in clear solution (chloral hydrate:water:glycerol, 8:2:1 [w/v/v]). The Cleared seeds were examined by using a microscope equipped with Nomarski optics.

## Supplementary Information

Supplementary Information.

## Data Availability

*Accession numbers for sequence data* PeMADS1 (AF234617), PeMADS7 (JN983500), PeSEP3 (KF673859), PeMADS28 (KT865880), AtsMADS29 (XP_020188803), TaWM25 (CAM59071), HvBsister (BAK06913), BdMADS29 (NP_001288325), OsMADS29 (XP_015624837), SbBsister (XP_002453370), ZMM17 (NP_001105130), OsMADS30 (Q655V4), OsMADS31 (Q84NC2), MaMADS29 (XP_018678849), PdAP3-like (XP_00880798), AcAP3-like (XP_020109780), EpMADS24 (AHM92100), VvFBP24 (RVW42148), VrFBP24-like (XP_034698718), ABS (Q8RYD9), RsTT16-like (XP_018481949), AmDEFH21 (CAC85225), FBP24 (AAK21255), SlFBP24-like (XP_019066630), AmtGGM13 (XP_006829168.2), CcBsister1 (ADD25185), GGM13 (CAB44459), GbMADS10 (BAD93174), GORDITA (NP_174399.2), CrAGL63 (XP_006306362.2), LcAGL63 (APB93359).
