# Supplementary material for: Orchid Bsister gene PeMADS28 displays conserved function in ovule integument development
Source: Sci Rep. 2021 Jan 13;11:1205. doi: 10.1038/s41598-020-79877-9 (PMC7806631; doi:10.1038/s41598-020-79877-9)
Supplement: Supplementary file 1 — Supplementary Information. [file 41598_2020_79877_MOESM1_ESM.docx]

**Orchid B_sister_ gene *PeMADS28* displays conserved function in ovule integument development**

Ching-Yu Shen^1†^, You-Yi Chen^2†^, Ke-Wei Liu^3,4^, Hsiang-Chia Lu^1,5,6^, Song-Bin Chang^2^, Yu-Yun Hsiao^7^, Fengxi Yang^8^, Genfa Zhu^8^, Shuang-quan Zou^5,9^, Lai-Qiang Huang^3,4^, Zhong-Jian Liu^5*^, and Wen-Chieh Tsai^1,2,7*^

**Content**

**Supplemental Figures**……………………………………………………………….………3

**Supplementary Figure 1 |**Alignment of amino acid sequence of PeMADS28 and other B_sister_ proteins………………………………………………………………………………….….…3

**Supplementary Figure 2 |**The expression patterns of the *PeMADS28* in various developmental stages of floral bud, floral organ and vegetative tissues of *P. equestris* by real-time RT–PCR analysis. …………………..………………………………………….……..…4

**Supplementary Tables**………………………………………………………………………..5

**Supplementary Table 1 |Table. S1.** List of primers used in this study…………...………….5

**Supplementary Figures**


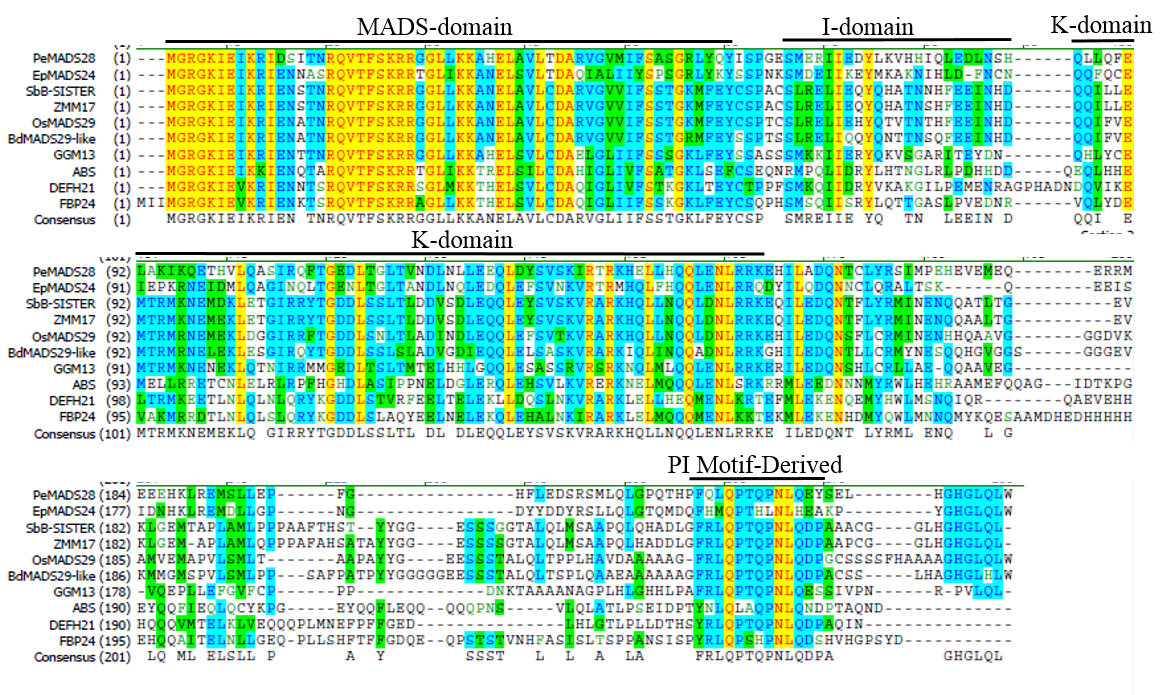


**Supplementary Figure 1 |Alignment of amino acid sequence of PeMADS28 and other B_sister_ proteins.**

Multiple sequence alignment with other monocot B_sister_ proteins demonstrated that PeMADS28 have a typical MIKC-type domain structure. The PI motif is highly conserved motif in their C-terminal regions that are also typical of B proteins.

**Supplementary Figure 2 |** **The expression patterns of the *PeMADS28* in various developmental stages of floral bud, floral organ and vegetative tissues of *P. equestris* by real-time RT–PCR analysis.** Quantification was normalized to *Phalaenopsis* *actin* for each sample. S1: stage 1 flower bud (0~2 mm); S2: stage 2 flower bud (2~4 mm); S3: stage 3 flower bud (4~6 mm); S4: stage 4 flower bud (6~8 mm); S5: stage 5 flower bud (8~10 mm);Se: sepal; Pe: petal; Li: lip; Co: column; P: pedicel; F: floral stalk; L: leaf; R: root. Error bars : ± SD (n=3 each).

**Supplementary Tables**

| **Supplementary Table 1 \| List of primers used in this study.** |
| --- |
| **RT-PCR** |
| PeMADS28: 5’-GGATCCATGGGGAGAGGGAAGATTG-3’ |
| 5’-GGATCCTTACCAGAGCTGCAGTCCA-3’ |
| **Quantitative real-time PCR** |
| PeMADS28: 5’-CTGGTGAAAGCATGGAAAGAATC-3’ |
| 5’-AATGCTCGCCTGAAGCACAT-3’ |
| ***In situ* hybridization probes** |
| PeMADS28: 5’-CCAGAGCATGAAGTGGAGAT-3’ |
| 5’-TGGAAGTGTTTGTGGGCCCA-3’ |
| **Subcellular localization and interaction analysis** |
| PeMADS28: 5’-GGGGACAAGTTTGTACAAAAAAGCAGGCTGG-3’ |
| 5’-GGGGACCACTTTGTACAAGAAAGCTGGGTT-3’-3’ |
| **Transformation in *Arabidopsis*** |
| PeMADS28: 5’-GGATCCATGGGGAGAGGGAAGATTG-3’ |
| 5’-GGATCCTTACCAGAGCTGCAGTCCA-3’ |
